# Investigating the feasibility of ^18^F‐flortaucipir PET imaging in the antemortem diagnosis of primary age‐related tauopathy (PART): An observational imaging‐pathological study

**DOI:** 10.1002/alz.14301

**Published:** 2024-10-17

**Authors:** Anna Lavrova, Ryota Satoh, Nha Trang Thu Pham, Aivi Nguyen, Clifford R. Jack, Ronald C. Petersen, Reichard R. Ross, Dennis W. Dickson, Val J. Lowe, Jennifer L. Whitwell, Keith A. Josephs

**Affiliations:** ^1^ Department of Radiology Mayo Clinic Rochester Minnesota USA; ^2^ Department of Laboratory Medicine and Pathology Mayo Clinic Rochester Minnesota USA; ^3^ Department of Neurology Mayo Clinic Rochester Minnesota USA; ^4^ Department of Neuroscience Mayo Clinic Jacksonville Florida USA

**Keywords:** ^18^F‐flortaucipir PET, definite PART, PART, primary age‐related tauopathy, tau‐PET

## Abstract

**INTRODUCTION:**

Primary age‐related tauopathy (PART) is characterized by neurofibrillary tangles and minimal β‐amyloid deposition, diagnosed postmortem. This study investigates ^18^F‐flortaucipir (FTP) PET imaging for antemortem PART diagnosis.

**METHODS:**

We analyzed FTP PET scans from 50 autopsy‐confirmed PART and 13 control subjects. Temporal lobe uptake was assessed both qualitatively and quantitatively. Demographic and clinicopathological characteristics and voxel‐level uptake using SPM12 were compared between FTP‐positive and FTP‐negative cases. Intra‐reader reproducibility was evaluated with Krippendorff's alpha.

**RESULTS:**

Minimal/mild and moderate FTP uptake was seen in 32% of PART cases and 62% of controls, primarily in the left inferior temporal lobe. No demographic or clinicopathological differences were found between FTP‐positive and FTP‐negative cases. High intra‐reader reproducibility (α = 0.83) was noted.

**DISCUSSION:**

FTP PET imaging did not show a specific uptake pattern for PART diagnosis, indicating that in vivo PART identification using FTP PET is challenging. Similar uptake in controls suggests non‐specific uptake in PART.

**Highlights:**

- ^18^F‐flortaucipir (FTP) PET scans were analyzed for diagnosing PART antemortem.
- 32% of PART cases had minimal/mild FTP uptake in the left inferior temporal lobe.
- Similar to PART FTP uptake was found in 62% of control subjects.
- No specific uptake pattern was found, challenging in vivo PART diagnosis.

## 1 BACKGROUND

Primary age‐related tauopathy (PART) is a neuropathological term used to describe cases in which tau‐positive Alzheimer's disease‐like neurofibrillary tangles (NFTs) are observed in the brain (Braak NFT stage ≤ IV) with minimal to absent β‐amyloid (Aβ) deposition.^1^, ^2^ Four PART categories are recognized: asymptomatic and symptomatic definite PART in which Aβ is absent (Thal phase 0), and asymptomatic and symptomatic possible PART in which Aβ is present but limited in distribution (Thal phase1‐2).^3^, ^4^ Definite PART is well accepted as a distinct entity, but there is overlap between possible PART and low Alzheimer's disease neuropathologic changes (ADNC),^5^, ^6^ which results in possible PART being a somewhat controversial entity. PART can be observed in relative isolation, or it can also present a co‐pathology with other neurodegenerative pathologies.

Currently, a diagnosis of PART can be rendered only through post mortem histological brain tissue examination and is independent of antemortem cognitive status.^1^ Since recent clinical trials for neurodegenerative diseases have focused on targeting pathological tau aggregates,^7^ the in vivo identification of the presence of tau proteins could advance the development of anti‐tau therapies by enabling appropriate subject selection, early intervention, and assessment of target engagement. To achieve these goals, ^18^F‐flortaucipir (FTP), a positron emission tomography (PET) tau protein‐ligand, was introduced.^8^, ^9^ Flortaucipir shows excellent binding to the paired helical filament of tau deposited as NFTs in individuals with AD,^10^, ^11^, ^12^, ^13^, ^14^ matches well with the severity and regional distribution of NFTs staged by Braak in AD,^15^, ^16^, ^17^, ^18^, ^19^ and exceeds other diagnostic modalities in differentiating AD from other neurodegenerative diseases.^20^

Tau‐PET research has evolved in AD, with many studies focusing on tau but in the presence of Aβ.^21^, ^22^, ^23^, ^24^, ^25^, ^26^ The literature on the application of tau‐PET imaging in autopsy‐confirmed PART is sparse. Studies assessing cognitively unimpaired cohorts have identified individuals with temporal lobe tau accumulation in the absence of Aβ deposition on PET, with tau uptake in these individuals related to cognition, and concluded that these individuals may have PART.^27^, ^28^, ^29^, ^30^, ^31^ However, these studies do not have autopsy confirmation of PART in cases with antemortem tau‐PET. In a small autopsy series from our institution, a temporal lobe meta‐region‐of‐interest (ROI) was unable to detect autopsy‐confirmed PART in three pure PART cases,^32^ as was the case in 12 of 13 PART cases that had additional neurodegenerative diseases in another study.^33^ The entorhinal cortex was also not found to be helpful in detecting PART in a large autopsy study from our institution.^19^ These findings suggest that FTP PET may not be sensitive to the NFT pathology of PART. It is possible, however, that the temporal lobe meta‐ROI and entorhinal cortex could miss subtle and focal uptake as well as uptake in lateral temporal lobe regions. Furthermore, unlike the handful of studies assessing the sensitivity of tau‐PET in PART, knowledge of the specificity of uptake in PART is limited.^19^

Our current study aimed to determine whether autopsy‐confirmed PART cases show uptake on FTP PET and, if so, how frequently, in which regions, and of what severity. We also aimed to determine whether uptake in PART is specific to the presence of NFTs or whether it is likely a result of off‐target binding. To address the limitations of studies using the meta‐ROI or just the entorhinal cortex, we performed a careful visual assessment of FTP uptake across individual temporal lobe regions, as well as a validation analysis using quantitative FTP uptake. We included only cases of definite PART, that is, a Thal Aβ phase 0 and Braak NFT stage of I‐IV, to eliminate any overlap/controversy with low ADNC. We also assessed uptake in temporal lobe regions in a control group of patients with no ADNC to assess specificity. Based on previous studies,^19^, ^34^, ^35^ we hypothesized that FTP would not show any specific pattern of uptake in individuals with definite PART. The findings of this study help better understand the relationship between FTP uptake and PART, as well as the feasibility of FTP PET imaging as a potential tool for in vivo PART diagnosis. 

**RESEARCH IN CONTEXT**

1. **Systematic review**: The authors reviewed existing literature on ^18^F‐flortaucipir (FTP) PET imaging for diagnosing primary age‐related tauopathy (PART). They focused on autopsy‐confirmed cases, comparing PET findings and clinicopathological data to assess FTP uptake patterns and diagnostic reliability.
2. **Interpretation**: Our analysis indicates that FTP PET imaging does not provide a specific uptake pattern for PART diagnosis. Both PART cases and controls showed minimal to moderate FTP uptake, primarily in the left inferior temporal lobe, with no significant demographic or clinicopathological differences. This non‐specific origin of uptake suggests that FTP PET is not a reliable biomarker for PART.
3. **Future directions**: Future research should seek specific PET tracers or biomarkers to differentiate PART from other tauopathies. Longitudinal studies on FTP uptake progression and its clinical correlation, combined with other imaging or fluid biomarkers, are necessary to improve diagnostic accuracy. Expanding sample sizes and including diverse populations will enhance the generalizability of the results.

## 2 METHODS

### 2.1 Study design and participants

We selected all individuals who had been enrolled in the Neurodegenerative Research Group (NRG), Mayo Clinic Alzheimer's Disease Research Center, or Mayo Clinic Study of Aging, who had undergone an antemortem FTP PET, and who had died and completed a brain autopsy examination (*n* = 248). Of these 248 cases, we selected all subjects with a Braak NFT stage I‐IV without any Aβ deposition (Thal phase 0) (*n* = 50). The fifty individuals with PART had died between April 8, 2016 and April 6, 2023, and included 15 females with a mean age of death of 74 years (± 11 years), range 53 to 104 years. Each participant's cognitive status was classified as dementia or not, based on Diagnostic and Statistical Manual of Mental Disorders (DSM) IV or DSM V criteria,^36^ and included assessment of global cognition via the Mini‐Mental State Examination.^37^

An additional group of control subjects with antemortem FTP PET and a Braak NFT stage = 0 without any Aβ deposition (Thal phase 0), who died between September 5, 2018 and October 10, 2023, was also identified (*n* = 13); this group comprised seven females and had a mean age of death of 64 years (± 10 years), ranging from 51 to 85 years.

### 2.2 Pathological and genetic analyses

All 63 cases and controls underwent a standardized pathological examination.^38^ Braak NFT staging^39^ used anti‐tau antibodies (AT8, 1:1,000 dilution), while the Thal phase^40^ utilized antibodies to Aβ (6F/3D, 1:10 dilution). Participants met definite PART criteria^2^ with low Braak NFT stages (I‐IV)^41^, ^42^ and no Aβ (Thal phase 0).^40^, ^43^, ^44^ Irrespective of clinical diagnosis, controls had Braak NFT stage 0 and Thal stage 0.

Consensus diagnostic criteria were utilized to determine the presence of frontotemporal lobar degeneration with tau (FTLD‐Tau), including corticobasal degeneration and progressive supranuclear palsy, or with TAR DNA binding protein 43 (FTLD‐TDP),^45^, ^46^ Lewy body disease,^47^ argyrophilic grain disease (AGD),^48^ and age‐related tau astrogliopathy (ARTAG).^49^ The presence of tau was determined using antibody CP13 (gift from the late Peter Davies), for Aβ (6F/3D; 1:10 dilution; Novocastra Vector Labs, Burlingame, CA); for TDP‐43 (pS409/410; mouse monoclonal; 1:5000; Cosmo Bio, Tokyo, Japan), and for α‐synuclein (EP1536Y; rabbit monoclonal; 1:40,000; Abcam, Waltham, MA). Immunohistochemistry was performed using IHC Autostainer 480S (Thermo Fisher Scientific Inc., Waltham, MA) and DAKO EnVision + reagents (Dako, Carpinteria, CA).

Genomic DNA extraction employed the QIAamp DNA mini kit for apolipoprotein E testing. The single‐day apolipoprotein E method^50^ was modified for amplification, followed by enzyme digestion and agarose gel electrophoresis.

### 2.3 Neuroimaging analysis

All participants underwent a structural head magnetic resonance imaging (MRI) and FTP PET. FTP PET scans were acquired using a PET/computed tomography (CT) scanner (GE Medical Systems, Milwaukee, Wisconsin) operating in 3D mode. An intravenous bolus injection of approximately 370 MBq (range 333 to 407 MBq) of FTP was administered, followed by a 20‐min PET acquisition performed 80 min after injection. Four 5‐min dynamic frames were acquired following a low‐dose CT transmission scan. Standard corrections were applied. Emission data were reconstructed into a 256 × 256 matrix with a 30‐cm field of view (FOV) (in‐plane pixel size = 1.17 mm). All participants also underwent a 3T head MRI protocol using GE scanners that included a magnetization‐prepared rapid gradient echo (MPRAGE) sequence (TR/TE/TI, 2300/3/900 ms; flip angle 8°, a 26‐cm FOV; 256 × 256 in‐plane matrix; phase FOV of 0.94; slice thickness of 1.2 mm).

### 2.4 Visual rating of FTP uptake

**FIGURE 1:**
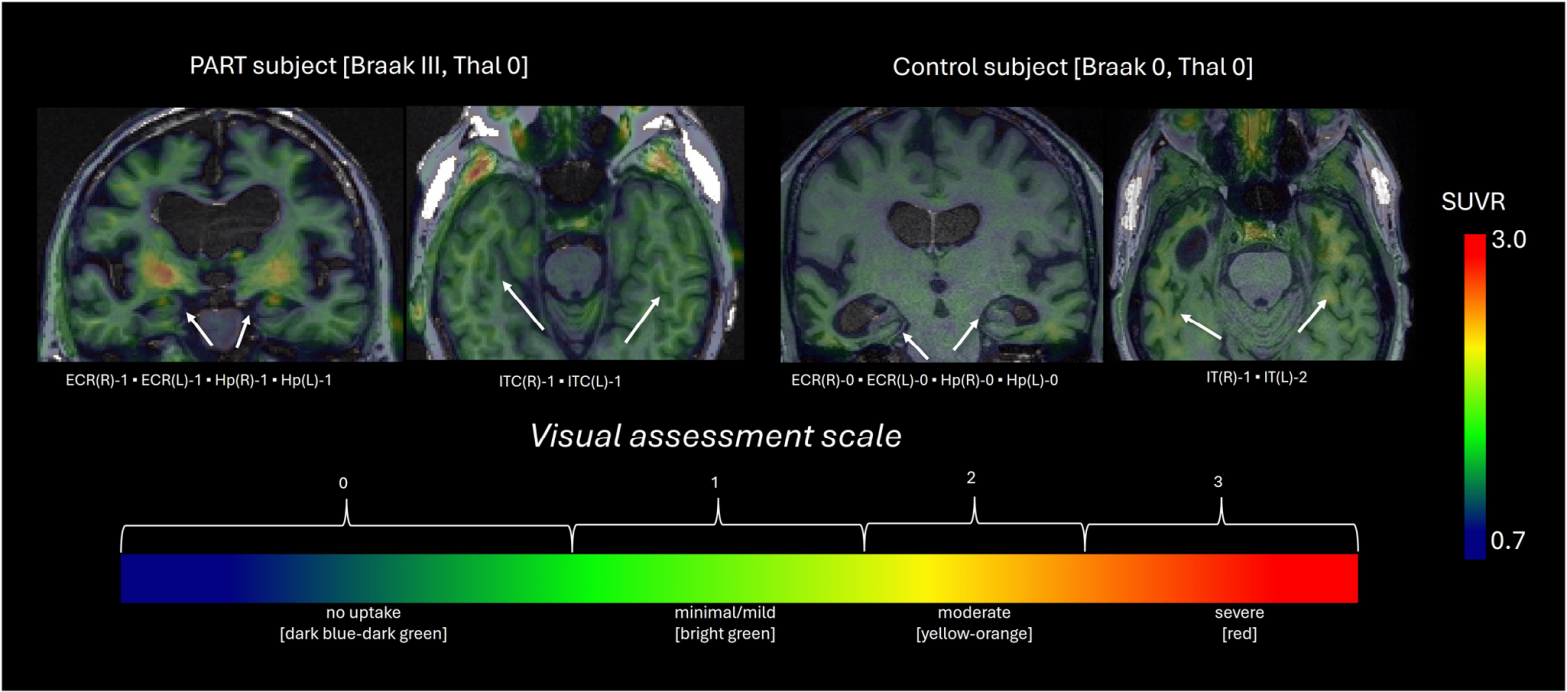
Visual grading scale used for ^18^F‐flortaucipir (FTP) PET images assessment. Upper row displays exemplary FTP‐PET scans in PART (Braak III/Thal 0) and control (Braak 0/Thal 0) subjects. Yellow arrows indicate regions assessed for FTP uptake. ECR, entorhinal cortex; Hp, hippocampus; ITC, inferior temporal cortex; L, left; PART, primary age‐related tauopathy; PET, positron emission tomography; R, right; SUVR, standardized uptake value ratio.

Individual‐level patterns of FTP uptake were evaluated using the FMRIB Software Library (FSL).^51^, ^52^, ^53^ The FTP PET images were co‐registered with the MPRAGE for better localization of the regions of interest. All voxels in the FTP images were divided by uptake in the cerebellar crus gray matter to create standardized uptake value ratio (SUVR) images. Flortaucipir uptake was scored by a radiologist (AL) using a visual assessment scale established before the analysis (Figure 1), with regions of interest selected to correspond to areas that have been reported to show uptake in Aβ‐negative individuals and to match Braak NFT stages of tau pathology: bilateral entorhinal cortex (Braak I), anterior hippocampus (Braak II), and inferior temporal cortex (Braak IV). FTP PET images were assessed in the ACTC (Brain colors) SUVR scale with brightness ranging from 0 to 3 and full image intensity; underlying MRI images were only used to navigate anatomy. FTP uptake was assessed using a simple, reproducible visual scale ranging from 0 to 3. A score of 0 indicated no uptake visualized, a score of 1 indicated minimal/mild uptake, a score of 2 indicated moderate uptake, and a score of 3 indicated severe/striking uptake. A case was classified as FTP‐positive if it scored 1 to 3 in any of the three regions, and a case was classified as FTP‐negative if it scored 0 in all three regions. To assess intra‐reader reproducibility, blinded to the previous scores, the same reader (AL) rated 20 randomly selected FTP PET scans from the same patient cohort using the same 0 to 3 grading scale after an approximately 2‐month gap between the evaluations.

### 2.5 Quantitative imaging analysis

The Mayo Clinic Adult Lifespan Template (MCALT) was used to output regional FTP uptake from the MPRAGE‐space FTP PET images for the entorhinal cortex, hippocampus, and inferior temporal cortex. Median values in these regions were divided by median uptake in the gray matter cerebellar crus to calculate the SUVR.

For voxel‐based analysis of FTP‐PET, the MPRAGE‐space SUVR images were transformed into MCALT template space using the parameters from the MPRAGE normalization and then smoothed using a Gaussian kernel with 6 mm FWHM. SPM12 was used to compare FTP‐positive and FTP‐negative cases in the PART cohort and between FTP‐positive PART and FTP‐positive controls. Results were assessed with age and gender as covariates and corrected for multiple comparisons using the family‐wise error (FWE) correction (*p* < 0.01).

### 2.6 Statistical analysis

Statistical analysis was performed using BlueSky Statistics version 10.3.1. Pearson's chi‐squared test was used to compare categorical variables, and the Kruskal–Wallis rank sum test was used for the comparison of continuous variables between FTP‐positive and FTP‐negative cases in the PART group. A *p*‐value of ≤0.05 was considered statistically significant. Krippendorff's alpha coefficient for categorical variables was calculated to assess intra‐reader reproducibility.

## 3 RESULTS

Of the 50 participants in this cohort with definite PART, 11 had isolated PART, and 39 had PART plus a co‐pathology.

### 3.1 Visual rating of FTP‐PET uptake

Sixteen FTP‐positive participants (32%) who exhibited FTP uptake in at least one of the three assessed regions were identified. Fourteen of these 16 PART cases had additional co‐pathology, and only two had isolated PART without co‐pathologies. The clinical, demographic, and pathological characteristics of the FTP‐positive and FTP‐negative cases in the PART cohort are shown in Table 1. No differences were found for the clinical, demographic, and pathological characteristics (*p* > 0.05).

**TABLE 1:** Demographic, clinical, and pathological features in FTP‐positive and negative cases in PART subjects.

	FTP‐negative (*N* = 34)	FTP‐positive (*N* = 16)	*p*‐value^a^
**Clinical and demographic characteristics**			
Cognitive impairment or dementia	23 (67.6%)	11 (68.8%)	0.94
MMSE (/30)	24.0 (16.2, 27)	26 (22, 29)	0.28
Age at death, years	75.1 (64.3, 83)	74.4 (68.4, 78.3)	0.69
Age at onset, years	57.8 (54.7, 65.6)	62.8 (58.2, 72.4)	0.21
Age at tau‐PET, years	71.6 (63.9, 80.9)	72.3 (64.7, 75.6)	0.52
Years from tau‐PET to death	1.5 (0.7, 2.9)	2.5 (1.0, 3.4)	0.2
Gender, F	10 (29.4%)	5 (31.2%)	0.89
Handedness, L/R	6/26	1/13	0.08
*APOE ε*4	4 (13.3%)	1 (6.2%)	0.46
**Pathological characteristics**			
AGD present	13 (38.2%)	4 (25.0%)	0.36
ARTAG present	10 (29.4%)	7 (43.8%)	0.32
^b^TDP‐43 present	3 (8.8%)	1 (6.2%)	0.75
Braak stage			0.66
I	8 (23.5%)	2 (12.5%)	
II	10 (29.4%)	5 (31.2%)	
III	12 (35.3%)	8 (50.0%)	
IV	4 (11.8%)	1 (6.2%)	
All FTLD (Tau + TDP) pathology	24 (70.6%)	8 (50.0%)	0.16
FTLD‐Tau (CBD)	8 (23.5%)	4 (25.0%)	0.91
FTLD‐Tau (PSP)	10 (29.4%)	2 (12.5%)	0.19
Isolated PART	9 (26.5%)	2 (12.5%)	0.27

*Note*: Data are shown as *N* (%) or median (Q1, Q3).

Abbreviations: AGD, argyrophilic grain disease; *APOE*, apolipoprotein E; ARTAG, age‐related tau astrogliopathy; CBD, corticobasal degeneration; FTP, ^18^F‐flortaucipir; FTLD, frontotemporal lobar degeneration; MMSE, Mini‐Mental State Examination; PART, primary age‐related tauopathy; PET, positron emission tomography; PSP, progressive supranuclear palsy; TDP‐43, trans‐active response DNA‐binding protein of 43 kDa.

^a^ Groupwise comparison *p* values are from Pearson's chi‐squared test and the Kruskal–Wallis rank sum test.

^b^ TDP‐43 may also be referred to as LATE_NC by some, but not all, groups of investigators.

A detailed description of FTP‐positive PART subjects is shown in Table 2. All 16 PART participants had uptake in the left inferior temporal cortex. Right inferior temporal cortex uptake was identified in nine of the 16 participants (56%); left entorhinal cortex and bilateral hippocampus uptake was observed in seven participants (44%); and right entorhinal cortex uptake was observed in six participants (38%). Flortaucipir uptake did not exceed a grade of 2 (moderate) in any region in any of the 16 individuals. Uptake followed no specific pattern other than being consistently observed in the left inferior temporal cortex. The remaining 34 cases were FTP‐negative. Of these 34 FTP‐negative cases, 16 had higher Braak stages of III or IV of which three cases had normal cognition at the time of death (Table S1). The Krippendorff's alpha coefficient on the categorical agreement between the first and second set of ratings on the same PART subjects from the same reader was *α* = 0.83 (almost perfect).

**TABLE 2:** Demographic and clinical features of the 16 PART cases with FTP uptake.

Subject #	Sex	Age at scan	Age at death	Illness duration (years)	*APOE* epsilon 4 genotype	Final clinical diagnosis	Pathological findings	Braak NFT stage	Regions showing FTP uptake	Years from scan to death
1	M	67.5	68.4	9.4	Negative	Corticobasal syndrome	FTLD‐tau (CBD), ARTAG	II	IT (L)	0.9
2^a^	M	75.4	76.3	–	Positive	Normal Cognition	–	II	IT (L)	0.9
3	M	81.2	82	4.3	Negative	PSP ‐Richardson syndrome	FTLD‐tau (PSP), AGD, ARTAG	II	IT (L), IT (R), Hp (R), Hp (L), EC (L)	0.8
4	M	71.4	74.5	16.0	Negative	FTD with parkinsonism	FTLD‐TDP type A, DLBD, rare Pre‐T in IT, HpScl	II	IT (L)	3.1
5	M	64.8	67.3	9.0	Negative	Dementia with Lewy bodies	DLBD	I	IT (L), IT (R), Hp (R), Hp (L), EC (L)	2.5
6	F	54.5	57.5	5.5	Negative	bvFTD	FTLD‐TDP type A, Pre‐ HpScl	I	IT (L)	2.9
7	M	74.9	77.8	5.9	Negative	PSP ‐Richardson syndrome	FTLD‐tau (CBD), AGD, ARTAG	III	IT (L), IT (R), Hp (R), Hp (L), EC (R), EC (L)	2.4
8	M	56.2	56.9	5.7	Negative	PSP ‐Richardson syndrome	FTLD‐tau (CBD), AGD, ARTAG	III	IT (L)	0.8
9	F	75.3	79.3	‐	Negative	Normal Cognition	TDP‐43	III	IT (L), IT (R), Hp (R), Hp (L), EC (R), EC (L)	4.1
10	M	82.6	85.1	–	Negative	Normal Cognition	iLBD	IV	IT (L), IT (R), Hp (R), Hp (L), EC (R), EC (L)	2.6
11	F	76.2	78	5.6	Negative	bvFTD	FTLD‐tau (CBD), AGD, ARTAG	III	IT (L)	1.8
12	M	67.7	73.6	10.8	Negative	SD	FTLD‐TDP type C, HpScl	III	IT (L), IT (R)	5.9
13	M	62.2	68.3	17.3	Negative	bvFTD	FTLD‐TDP type A, HpScl	II	IT (L), IT (R), Hp (R), EC (R)	6.1
14	F	64.4	68.5	10.3	Negative	SD	FTLD‐TDP type C	III	IT (L), IT (R), Hp (R), Hp (L), EC (R), EC (L)	4.2
15	M	73.2	74.3	6.3	Negative	Dementia with Lewy bodies	DLBD	II	IT (L), IT (R)	1.1
16	F	78.8	80.2	5.3	Negative	PSP—Richardson syndrome	FTLD‐tau (PSP), AGD, ARTAG	III	IT (L), Hp (L), EC (L)	1.5

Abbreviations: AGD, argyrophilic grains disease; *APOE*, apolipoprotein E; ARTAG, age‐related tau astrogliopathy; bvFTD, behavioral variant frontotemporal dementia; CBD, corticobasal degeneration; DLBD, diffuse Lewy body disease; EC, entorhinal cortex; FTLD, frontotemporal lobar degeneration; FTP, ^18^F‐flortaucipir; Hp, hippocampus; HpScl, hippocampal sclerosis; iLBD, incidental Lewy body disease; IT, inferior temporal; L, left; NFT, neurofibrillary tangle; PART, primary age‐related tauopathy; Pre‐T in IT, pretangles found in IT; PSP, progressive supranuclear palsy; R, right.; SD, semantic dementia; TDP‐43, trans‐active response DNA‐binding protein of 43 kDa.

^a^ Pretangles found in IT. *TDP‐43 may also be referred to as LATE_NC by some groups of investigators.

Of the 13 control cases, eight (62%) exhibited FTP uptake in at least one of the three assessed regions (Figure 1 and Table S2). There were no differences in demographic, clinical, pathological, or genetic characteristics between the positive and negative cases. In the group of eight FTP‐positive controls, all had uptake in the bilateral inferior temporal cortex (*n* = 8, 100%). Bilateral entorhinal cortex and left hippocampus uptake was observed in five participants (63%), and right hippocampus uptake was found in four participants (50%). Flortaucipir uptake did not exceed the grade of 2 (moderate) in any region in any of the eight individuals and no specific pattern other than the consistently observed uptake in the bilateral inferior temporal cortex was noted.

### 3.2 Quantification of FTP‐PET uptake

**FIGURE 2:**
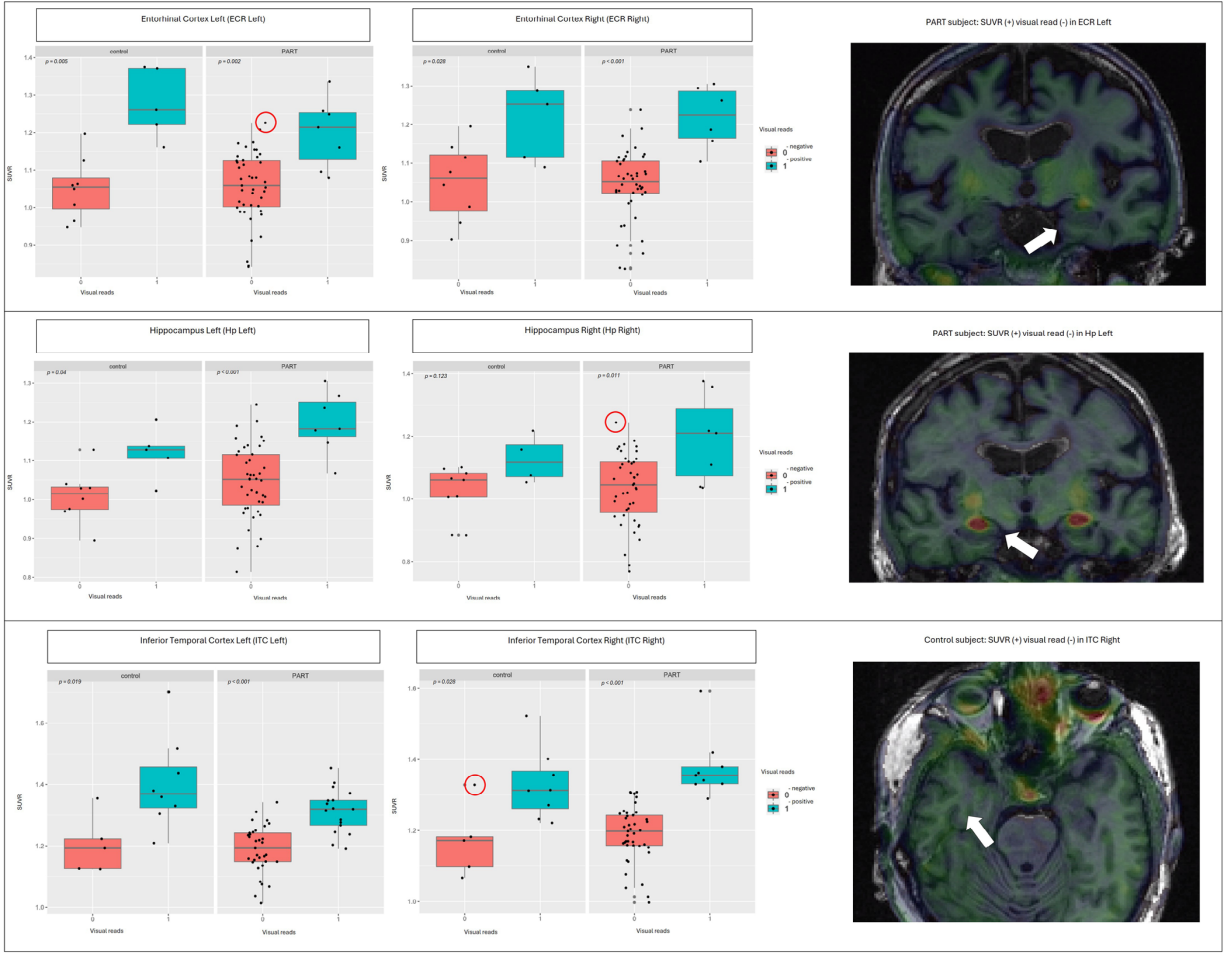
Boxplots display the standardized uptake value ratios (SUVRs) in ^18^F‐flortaucipir (FTP) PET for each region, separated by negative and positive visual reads. The right column presents exemplary cases with mismatches between quantitative and qualitative outputs, corresponding to the case highlighted by a red circle on the graph. *p*‐values indicating the differences between quantitative outputs were derived from the Kruskal–Wallis test. PART, primary age‐related tauopathy; PET, positron emission tomography.

In addition to the visual assessment of FTP PET scans, SUVRs for each region were compared between positive and negative visual reads in the PART and control groups. Significant differences in SUVRs were found in all assessed regions except for the right hippocampus in the control group (*p* = 0.123) (Figure 2).

**FIGURE 3:**
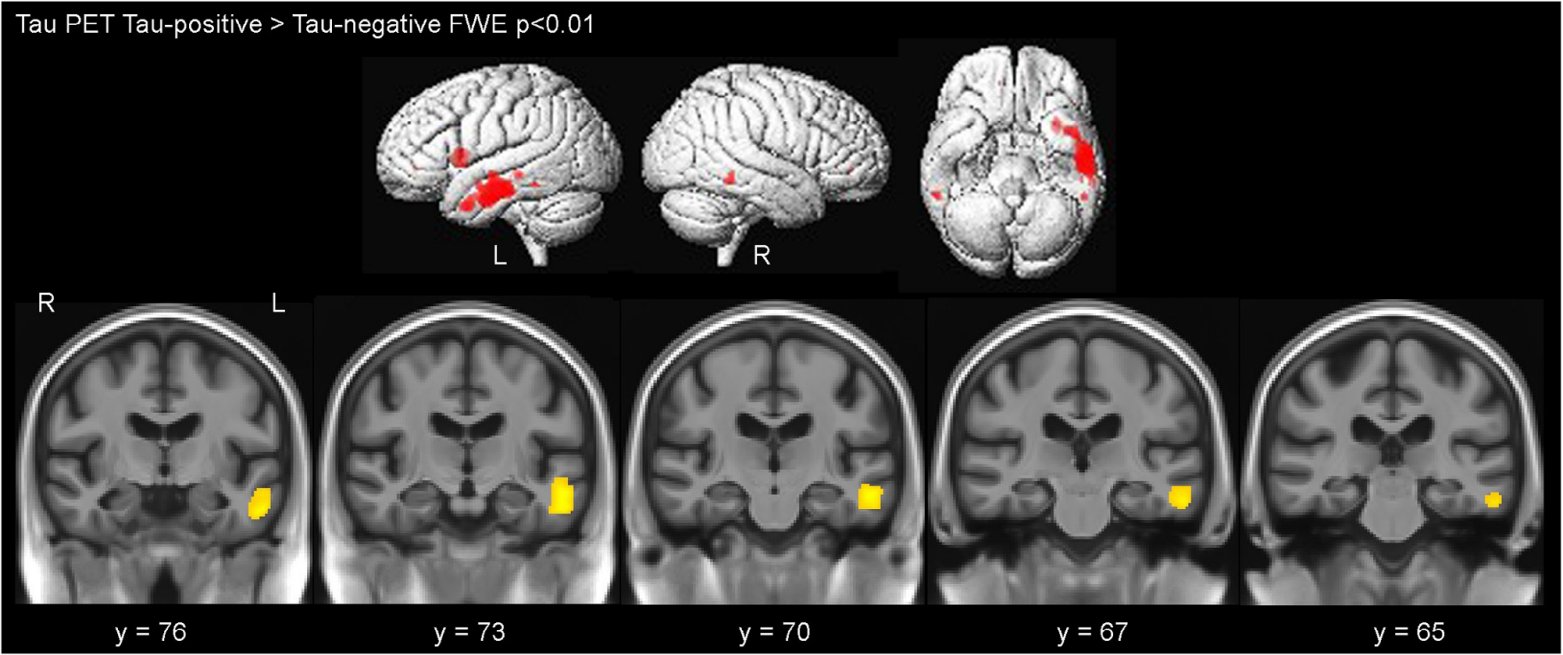
SPM12 analysis showing FTP uptake in FTP‐positive cases compared to FTP‐negative cases in a PART group. FTP, ^18^F‐flortaucipir; FWE, family‐wise error; PART, primary age‐related tauopathy; PET, positron emission tomography.

A voxel‐wise (SPM12) image analysis was used to depict differences between FTP‐positive PART and FTP‐negative PART cases (Figure 3) and between FTP‐positive PART and FTP‐positive controls. Greater FTP uptake was identified in the left inferior and middle temporal gyri white matter in the FTP‐positive PART cases compared to the FTP‐negative PART cases. Voxel‐wise comparison between FTP‐positive PART cases and FTP‐positive control cases showed no significant difference in uptake in either direction.

## 4 DISCUSSION

The findings from this study suggest that FTP PET is not a good biomarker for an antemortem diagnosis of definite PART. No specific uptake patterns helped identify PART cases on PET, and only 16 individuals (32%) had “non‐specific” ligand uptake found primarily in the left inferior temporal lobe, suggesting that PART diagnosis is challenging for in vivo identification. Upon comparison of FTP‐positive and negative cases in the PART cohort, no significant differences were found, showing that uptake in PART may be non‐specific to underlying tau pathology, at least for the fibrillary tau pathology in NFTs.

For the visual gradings, we implemented a grading scale that focuses on the medial and inferior temporal lobes, following the spread pattern of NFTs, to most precisely identify uptake that we might observe in PART. This scale differs from two other existing scales designed primarily for assessing ADNC rather than PART, where the spread of NFT is more widespread and possibly related to the presence of Aβ.^17^, ^54^ There are no data to suggest that either of these scales is superior to the other, and neither has been utilized or validated in PART.

The exact reason for higher uptake in some PART cases is still being determined. One possibility is that it reflects a higher NFT density or maturity in the FTP‐positive cases, assuming a higher sensitivity of neuroimaging to detect middle/advanced NFT maturity markers. Indeed, FTP uptake has similarly been shown to be greater in cortical regions of individuals with AD.^27^, ^55^, ^56^ Though similar to AD, NFTs in PART were reported to have a much greater abundance of extracellular (ghost) tangles than intracellular NFTs or pretangles, demonstrating preferential 3R immunoreactivity with uniform staining of lesser intensity than intracellular NFTs.^57^, ^58^ Developing reliable methods to distinguish between PART and AD NFTs, such as through biochemical or immunohistochemical markers, would help to address this issue.^59^ Another compelling argument against FTP detecting NFTs in PART is the observation that in some individuals, only the inferior temporal cortex shows uptake, while the entorhinal cortex remains unaffected. This contradicts the theory of NFT spread in the Braak staging system, which posits that the entorhinal cortex is affected before the temporal cortex.^60^ Furthermore, control cases without ADNC were analyzed and exhibited similar binding patterns to those with PART, suggesting that uptake may reflect binding to other non‐NFT targets. Indeed, uptake was observed predominantly in the temporal lobe white matter in the voxel‐based analysis, which may support this theory.

Other pathologies were present in 14 of the 16 FTP‐positive cases, including progressive supranuclear palsy (PSP), corticobasal degeneration (CBD), FTLD‐TDP, ARTAG, and AGD. However, the frequency of these additional pathologies did not differ between the FTP‐positive and FTP‐negative groups, suggesting that their presence is not driving the group differences. However, we could be observing a phenomenon whereby FTP‐positive PART cases with these co‐pathologies may have a higher burden of these co‐pathologies. The main couter argument for this statement is that autoradiographic studies have not found binding of FTP to these co‐pathologies.^10^ Another possibility is that the severity of co‐pathology–related neurodegeneration could be different between cases showing FTP uptake versus those without uptake.

Of the 50 PART participants in this study, the majority had a co‐pathology, with only 11 participants having PART in isolation. Only two of these 11 isolated PART cases showed some FTP uptake in the assessed regions. This frequency of approximately 18% is lower when compared to the frequency with some uptake in those with a co‐pathology (37.5%). Therefore, individuals with a co‐pathology and PART are twice as likely to show FTP uptake as those with primary or isolated PART. Another variable requiring some discussion is that of dementia. Most individuals from our cohort were cognitively impaired (*n* = 34, 68%). However, there was no difference in the number of cognitively impaired/unimpaired participants between the FTP‐positive and negative groups in the PART cohort, suggesting that uptake was unrelated to cognitive dysfunction.

One of the study's most interesting findings was a higher uptake observed in the controls. This finding suggests the likely non‐specificity of the uptake in PART and supports the result of our other study where we focused on the entorhinal cortex.^19^ We need to understand what FTP is binding to in the controls and why some cases exhibit uptake while others do not. It could also be due to the severity of pathology‐related neurodegeneration, as discussed above for PART. We also wonder whether some uptake could be associated with binding to immature tau, such as non‐fibrillary tau in the form of pretangles. Pretangles do not count toward a Braak NFT stage and may be present in cases with a Braak NFT stage of 0. Supporting this hypothesis is the observation that in the FTP‐positive PART Case 2, with uptake noted in the inferior temporal lobe, there was pathological evidence of pretangles in the inferior temporal lobe. A comprehensive pathological assessment of regional burden and distribution of pretangles, while adjusting for regional NFT burden and correlating with FTP uptake, is needed.

Our study has several strengths. First, we had a large cohort of autopsy‐diagnosed definite PART as well as a cohort of participants without NFTs or Aβ pathology. Second, the time from FTP scan to death was relatively short. Third, all pathological diagnoses were completed by a board‐certified neuropathologist with expertise in the diagnosis of AD and PART. Our study also has several limitations. First, we have a relatively small sample of primary or isolated PART cases, which is typically how PART is regarded in the field. Many individuals in our cohort had an FTLD spectrum disorder and other co‐pathologies, such as AGD and ARTAG, which often co‐exist with FTLD‐tau. Hence, our results might not fully reflect what the field considers as PART. Furthermore, many of our subjects were cognitively impaired, a situation similar to that of our control cohort. Another limitation is that we did not assess tau burden quantitatively, and Braak NFT staging does not account for the burden of regional tau pathology. Therefore, it is possible that, although the Braak stage did not differ between the PART positive and negative cases, the burden of NFTs (and hence tau) could differ. That said, the NFT burden does not explain the uptake observed in our control cases where NFTs were absent; burden of pretangles could differ however.

Our study has clinical and research implications. Given that recent clinical trials for neurodegenerative diseases have focused on targeting pathological tau aggregates, the availability of in vivo biomarkers that can detect and quantify tau protein has substantial diagnostic and clinical value. Our research shows that FTP PET does not reliably help to identify individuals with definite PART.

## CONFLICT OF INTEREST STATEMENT

Anna Lavrova, Ryota Satoh, Nha Trang Thu Pham, Aivi Nguyen, and Reichard R. Ross: no conflicts of interest. Dennis W. Dickson, Jennifer L. Whitwell, and Keith A. Josephs: NIH/NIA/NINDS/NIDCD research support. Clifford R. Jack, Jr.: Roche data monitoring board; Eisai speaker; Biogen consultant; NIH, GHR Foundation, and the Mayo Clinic research support. Ronald C. Petersen: Janssen Alzheimer Immunotherapy data monitoring committee member; Biogen, Roche, Merck, and Genentech, Inc. consultant; Oxford University Press royalties; NIH/NIA research support. Val J. Lowe: Bayer Schering Pharma, Piramal Life Sciences, Eisai Inc., AVID Radiopharmaceuticals, and Merck Research consultants; GE Healthcare, Siemens Molecular Imaging, AVID Radiopharmaceuticals, and the NIH (NIA, NCI) research support. Author disclosures are available in the supporting information.

## CONSENT STATEMENT

This study has been approved by the Mayo Clinic institutional review board, and all patients or their proxies signed a written informed consent form before taking part in any research activities in accordance with the Declaration of Helsinki.

## Supporting information



Supporting information

Supporting information
