# Supplementary material for: Investigating the feasibility of 18F‐flortaucipir PET imaging in the antemortem diagnosis of primary age‐related tauopathy (PART): An observational imaging‐pathological study
Source: Alzheimers Dement. 2024 Oct 17;20(12):8605–14. doi: 10.1002/alz.14301 (PMC11667508; doi:10.1002/alz.14301)
Supplement: Supplementary file 1 — Supporting information [file ALZ-20-8605-s001.docx]

**Supplemental Table 1.** Demographic and clinical features of the Braak III and IV PART cases without FTP uptake.

| **Subject #** | **Sex** | **Age at scan** | **Age at death** | **Illness duration (yrs.)** | **APOE epsilon 4 genotype** | **Final Clinical diagnosis** | **Pathological findings** | **Braak NFT stage** | **Years from scan to death** |
| --- | --- | --- | --- | --- | --- | --- | --- | --- | --- |
| 1 | M | 86 | 87.3 | - | Positive | Normal cognition | AGD, ARTAG,  TDP-43 | III | 1.4 |
| 2 | M | 83 | 83.8 | 11 | Positive | PPAOS | FTLD-tau (PSP) (atypical) | III | 0.9 |
| 3 | F | 82.9 | 83.6 | - | Negative | Normal cognition | AGD,  TDP-43 | III | 0.7 |
| 4 | M | 83.5 | 88.3 | 7.0 | Negative | PSP-Parkinsonism | FTLD-tau (PSP), TDP-43 | IV | 4.8 |
| 5 | F | 71.7 | 74.9 | 6.3 | Positive | PSP-Richardson syndrome | FTLD-tau (PSP) | III | 3.3 |
| 6 | M | 58.5 | 59.6 | 10.7 | Negative | PPAOS | FTLD-tau (CBD), AGD, ARTAG | IV | 1.2 |
| 7 | M | 67.9 | 75.5 | 15.5 | Negative | Dementia with Lewy bodies | DLBD,  ARTAG | III | 7.6 |
| 8 | M | 78.2 | 79.6 | 11.0 | Negative | PSP-Richardson syndrome | FTLD-tau (PSP) (atypical) | IV | 1.5 |
| 9 | M | 74.7 | 75.3 | 6.6 | Negative | AOS-PAA | FTLD-tau (CBD) | III | 0.6 |
| 10 | M | 67.4 | 68.9 | 6.1 | Negative | PPAOS | FTLD-tau (CBD), AGD | III | 1.5 |
| 11 | M | 71.6 | 74.1 | 10.1 | Negative | PSP-Richardson syndrome | FTLD-tau (PSP), ARTAG | III | 2.6 |
| 12 | F | 65.9 | 67 | 3.5 | Negative | PSP-Richardson syndrome | FTLD-tau (CBD) | III | 1.2 |
| 13 | F | 60.8 | 63.5 | 6.8 | Negative | PSP-Richardson syndrome | FTLD-tau (PSP) | III | 2.7 |
| 14 | M | 51.4 | 52.9 | 2.6 | Negative | Corticobasal syndrome | FTLD-tau (CBD), AGD, ARTAG | IV | 1.5 |
| 15 | M | 60 | 63.1 | 5.3 | Negative | PSP-Richardson syndrome | FTLD-tau (PSP), ARTAG | III | 3.1 |
| 16 | M | 79.2 | 81 | - | Negative | Normal cognition | ARTAG | III | 1.79 |

*Abbreviations:* AGD = argyrophilic grain disease, AOS-PAA = apraxia of speech-progressive agrammatic aphasia, APOE = apolipoprotein; ARTAG = aging-related tau astrogliopathy, CBD = corticobasal degeneration, DLBD = diffuse Lewy body disease; FTLD = frontotemporal lobar degeneration; HpSc = hippocampal sclerosis; PPAOS = primary progressive apraxia of speech; PSP = progressive supranuclear palsy.

**Supplemental Table 2.** Demographic and clinical features of the control cases (Braak 0, Thal 0) with and without FTP uptake.

| **Subject #** | **Sex** | **Age at scan** | **Age at death** | **Illness duration (yrs.)** | **APOE epsilon 4 genotypes** | **Final Clinical diagnosis** | **Pathological findings (brain)** | **Years from scan to death** | **Regions showing FTP uptake** | **Cause of death** |
| --- | --- | --- | --- | --- | --- | --- | --- | --- | --- | --- |
| 1 | M | 82 | 84.7 | - | Negative | Normal cognition | Rare pretangles | 2.7 | IT (L), IT (R), Hp (L), Hp (R), EC (L), EC (R) | Metastatic melanoma |
| 2 | F | 72 | 73.9 | 7.5 | Negative | bvFTD | FTLD-tau (CBD), AGD, ARTAG, TDP-43 | 1.9 | - | Neurodegenerative disease |
| 3 | F | 67 | 69.1 | 4.7 | Negative | bvFTD, PPA | FTLD-TDP type A | 2.1 | - | Neurodegenerative disease |
| 4 | F | 50 | 50.7 | - | Negative | Normal cognition | - | 0.7 | - | Cardiac arrhythmia |
| 5 | F | 64 | 67.5 | 8.3 | Negative | bvFTD | FTLD-tau (CBD), ARTAG  TDP-43 | 3.5 | IT (L), IT (R) | Neurodegenerative disease |
| 6 | F | 72 | 75.8 | 12.2 | Negative | SD | FTLD-tau (PiD) | 3.8 | IT (L), IT (R), Hp (L), Hp (R), EC (L), EC (R) | Neurodegenerative disease |
| 7 | F | 51 | 53 | 5.1 | Negative | AOS-PAA | FTLD-tau (PiD) | 2 | IT (L), IT (R), Hp (L), Hp (R), EC (L), EC (R) | Neurodegenerative disease |
| 8 | M | 60 | 60.9 | - | Negative | Normal cognition | - | 0.9 | - | Accident |
| 9 | M | 53 | 55.8 | - | Positive | Normal cognition | - | 2.8 | - | Cirrhosis |
| 10 | M | 66 | 68.4 | 4.7 | Negative | PSP | FTLD-tau (PSP) | 2.4 | IT (L), IT (R) | Neurodegenerative disease |
| 11 | M | 53 | 57.9 | 12.0 | Negative | SD, rtvFTD | FTLD-TDP type C | 4.9 | IT (L), IT (R), Hp (L), Hp (R), EC (L), EC (R) | Neurodegenerative disease |
| 12 | M | 56 | 61.2 | 8.5 | Negative | SD, rtvFTD | FTLD-tau (GGT) | 5.2 | IT (L), IT (R), Hp (L), Hp (R), EC (L), EC (R) | Neurodegenerative disease |
| 13 | F | 54 | 56.6 | 5.0 | Negative | bvFTD | FTLD-tau (CBD) | 2.6 | IT (L), IT (R) | Neurodegenerative disease |

*Abbreviations:* AOS-PAA = apraxia of speech-progressive agrammatic aphasia, ARTAG = aging-related tau astrogliopathy, AGD = argyrophilic grain disease, bvFTD = behavioral variant frontotemporal dementia, CBD = corticobasal degeneration, FTLD-tau = frontotemporal lobar degeneration with tau pathology, GGT = globular glial tauopathy, PiD = Pick’s disease; PPA = primary progressive aphasia, PSP = progressive supranuclear palsy, rtvFTD = right temporal variant frontotemporal dementia, SD = semantic dementia.
